# Public Health Communication in Time of Crisis: Readability of On-Line COVID-19 Information

**DOI:** 10.1017/dmp.2020.151

**Published:** 2020-05-11

**Authors:** Corey H. Basch, Jan Mohlman, Grace C. Hillyer, Philip Garcia

**Affiliations:** Department of Public Health, William Paterson University, Wayne, New Jersey; Department of Psychology, William Paterson University, Wayne, New Jersey; Department of Epidemiology, Mailman School of Public Health, Columbia University, New York, New York

**Keywords:** COVID-19, on-line information, readability

## Abstract

**Objective:**

The purpose of this study was to assess the readability of information on the Internet posted about coronavirus disease 2019 (COVID-19) to determine how closely these materials are written to the recommended reading levels.

**Methods:**

Using the search term “coronavirus,” information posted on the first 100 English language websites was identified. Using an online readability calculator, multiple readability tests were conducted to ensure a comprehensive representation would result.

**Results:**

The mean readability scores ranged between grade levels 6.2 and 17.8 (graduate school level). Four of the 5 measures (GFI, CLI, SMOG, FRE) found that readability exceeded the 10th grade reading level indicating that the text of these websites would be difficult for the average American to read. The mean reading level for nearly all noncommercial and commercial websites was at or above the 10th grade reading level.

**Conclusions:**

Messages about COVID-19 must be readable at an “easy” level, and must contain clear guidelines for behavior. The degree to which individuals seek information in response to risk messages is positively related to the expectation that the information will resolve uncertainty. However, if the information is too complex to interpret and it fails to lead to disambiguation, this can contribute to feelings of panic.

Coronavirus disease 2019 (COVID-19) is caused by the virus severe acute respiratory syndrome coronavirus-2 (SARS-CoV-2), and, as of the end of March 2020, is responsible for nearly half a million cases and over 20,000 deaths in 200 countries, areas, or territories.^1^ Thus, the World Health Organization declared this a pandemic.^1^ As information about this pandemic is slowly unfolding, and while guidelines and restrictions are being formulated and distributed, the general public is left to seek information to inform their health decisions.

Written communication is an essential tool in times of crisis; thus, it is imperative to understand optimal elements of emergency messages. Research to date suggests that written messages are more easily and accurately remembered than auditory messages.^2^ Regardless of mode of transmission, messages pertaining to risk must contain accurate information, be rapidly disseminated, and be easily understood by the majority of the population.^2,3^ Health professionals recommend that materials for the general public, particularly in emergencies, be readable at the 6th grade reading level to have maximum impact,^4^ yet there are no published studies that analyze the readability of information on COVID-19. To this end, the purpose of this study was to assess the readability of information on the Internet posted about COVID-19 to determine how closely these materials are written to the recommended reading levels.

## METHODS

The methods for this study were based on a prior cross-sectional study assessing readability.^5^ Using the keyword “coronavirus,” a search of the Internet using Google Chrome as a browser was conducted. Websites were vetted to ascertain that they contained relevant content written in English. The sample was comprised of information from the first 100 websites that met the inclusion criteria resulting from the search. Articles were included if they were written in the English language, contained material relevant to COVID-19, and had a distinct URL leading to an article as opposed to a “splash” or menu page. Articles were excluded if they were derived from a sponsored website.

All URL extensions and websites were recorded, and were processed using on-line readability software, Readable.io.^6^ We conducted 5 readability tests to ensure a comprehensive representation would result. Those readability tests were as follows: Coleman-Liau Index (CLI), Gunning Fog Index (GFI), the Simple Measure of Gobbledygook (SMOG) Grade Level, Flesch-Kincaid Grade Level (FKGL), and Flesch-Kincaid Reading Ease (FRE). Each test measures the same construct, readability, but does so in slightly different ways.^6^ CLI is based on the average sentence length and an average of the number of letters for every 100 words.^6^ The GFI assesses the frequency of words that are polysyllabic in conjunction with an average of the length of sentences.^6^ The SMOG test also assesses the frequency of words that are polysyllabic but does so in a sample of sentences.^6^ The FKGL and FRE tests both involve determining the mean number of syllables per word and the mean sentence length.^6^

Readability measures reporting US grade levels were then categorized as “easy,” “average,” and “difficult,” corresponding to less than grade 6, grades 6-10, and greater than grade 10 reading levels, respectively. The mean readability score and standard deviation were calculated for each measure along with the range of minimum and maximum scores. Based on the URL extension, websites were then categorized at commercial (.com and.net) or noncommercial (.org,.gov,.edu). Comparisons between the mean score for each measure were computed using analysis of variance and between grade level categories (easy, average, and difficult) for commercial versus noncommercial websites using chi square test or Fisher’s exact test. *P*-Values <0.05 were considered statistically significant. All analyses were conducted using IBM SPSS version 26. The Institutional Review Board at William Paterson University does not review studies that do not include human subjects.

## RESULTS

For the 100 websites examined, the mean readability scores ranged between grade levels 6.2 and 17.8 (graduate school level). Four of the 5 measures (GFI, CLI, SMOG, FRE) found that readability exceeded the 10th grade reading level, indicating that the text of these websites would be difficult for the average American to read (Table 1). Only 4 websites were scored as “easy, <6th grade” (GFI). The mean reading level for nearly all noncommercial and commercial websites was at or above the 10th grade reading level with the exception of commercial URLs scored with the FKGL (9.9; SD 1.7) (Table 2). Only when using the CLI measure was a difference between website type detected with noncommercial scored at the 12.2 grade level versus commercial at 11.5 (*P* = 0.04). When evaluating noncommercial and commercial websites by easy, average, and difficult readability categories, the SMOG detected a borderline significant difference with 60.4% of noncommercial sites coded as difficult versus 78.7% of commercial (*P* = 0.048); no differences were detected with the other 4 measures.

**TABLE 1:** Readability Characteristics of Coronavirus Websites

	Number of websites (*n* = 100)			Readability Score	
Test	Easy (Grade <6)	Average (Grade 6-10)	Difficult (Grade >10)	Mean [SD]	Range
FKGL	0	68	32	10.0 [1.9]	6.2 – 15.4
GFI	4	47	49	10.5 [2.8]	0.9 – 16.1
CLI	0	32	68	11.9 [1.8]	6.0 – 16.2
SMOG	0	31	69	11.9 [1.8]	7.6 - 17.8
FRE^a^	0	9	91	46.4 [11.1]	14.9 – 65.0

a FRE scored on a scale of 0-100, with higher scores indicating greater ease of readability.

**TABLE 2:** Comparison of Coronavirus Websites by URL Extension (Non-commercial vs. Commercial), n = 100

	Mean [SD]			Non-commercial URL (*n* = 53)			Commercial URL (*n* = 47)			
	Non-commercial URL (*n* = 53)	Commercial URL (*n* = 47)		Easy (Grade <6)	Average (Grade 6-10)	Difficult (Grade >10)	Easy (Grade <6)	Average (Grade 6-10)	Difficult (Grade >10)	
Test			*P*-Value	*N* (%)	*N* (%)	*N* (%)	*N* (%)	*N* (%)	*N* (%)	*P*-Value
FKGL	10.1 [2.1]	9.9 [1.7]	0.63	0 (0.0)	36 (67.9)	17 (32.1)	0 (0.0)	32 (68.1)	15 (31.9)	0.99
GFI	10.2 [3.1]	10.9 [2.4]	0.25	2 (3.8)	27 (50.9)	24 (45.3)	2 (4.3)	20 (42.6)	25 (53.2)	0.70
CLI	12.2 [1.9]	11.5 [1.6]	0.04	0 (0.0)	14 (26.4)	39 (73.6)	0 (0.0)	18 (38.3)	29 (61.7)	0.20
SMOG	12.0 [2.0]	11.9 [1.6]	0.85	0 (0.0)	21 (39.6)	32 (60.4)	0 (0.0)	10 (21.3)	37 (78.7)	0.048
FRE^a^	44.1 [12.5]	49.0 [8.7]	0.03	0 (0.0)	6 (11.3)	47 (88.7)	0 (0.0)	3 (6.4)	44 (93.6)	0.50

a FRE scored on a scale of 0-100 with higher scores indicating greater ease of readability.

## DISCUSSION

The material on websites analyzed in this study was written at reading levels much higher than recommended. Given that negative emotional states may function as an obstacle to the readability of messages, materials written at higher than recommended levels can further compound the issue. Following the announcement of a crisis, such as the outbreak of a pandemic-type illness, anxiety would be expected to rapidly rise in the general public.^7^ Anxiety is known as the “fight or flight” syndrome and, as such, is meant to gear the mind and body up for action.^8^ The body becomes energized to cope with threat, and attention becomes narrowly focused on threat-related information, sometimes to the detriment of accurate interpretation or assimilation of information. Thus, anxiety acts both as a catalyst for information seeking and an obstacle to the ability to read and interpret written messages,^9^ particularly when prior knowledge of the subject is limited.^10^ Thus, anxiety fuels the quest for disambiguating information in poorly understood emergency situations, and when none is found or when the message itself is difficult to comprehend, anxiety is likely to rise.^11^

This cycle of anxiety-fueled information seeking, difficulty comprehending health related information, or discovery of a lack of disambiguating information, can then fuel panic and lead to maladaptive behaviors, such as unnecessary trips to emergency rooms or overuse of other emergency health resources.^12,13^ Thus, the cascade of events in response to anxiety provoking messages is associated with strain on resources.^12,13^

Thus, we argue that messages about COVID-19 must be readable at an “easy” level, and must contain clear guidelines for behavior. The degree to which individuals seek information in response to risk messages is positively related to the expectation that the information will resolve uncertainty.^9^ However, if the information is too complex or too challenging to interpret and it fails to lead to disambiguation, this can contribute to feelings of anxiety and panic.

This study is limited as it represents websites at a single cross-section of time. As the Internet is constantly being updated with new information, especially in an evolving situation, the search would likely yield different results at a later point in time. It is possible that messages are tailored by authors to specific types of audiences, and we did not assess audience type.^14^ The readability tools are capable of text analysis only, and do not provide any assessment of graphics that may be housed on the pages. Furthermore, the limitation to information in English only does not offer insight beyond 1 language. Nevertheless, this research fills a gap in literature and demonstrates the need for those communicating vitally important information in a time of crisis to be more diligent in writing content that can be easily understood by the largest number of individuals.
